# Decreased Expression of DREAM Promotes the Degeneration of Retinal Neurons

**DOI:** 10.1371/journal.pone.0127776

**Published:** 2015-05-28

**Authors:** Shravan Chintala, Mei Cheng, Xiao Zhang

**Affiliations:** Laboratory of Ophthalmic Neurobiology, and Eye Research Institute of Oakland University, Rochester, Michigan, United States of America; Boston University School of Medicine, UNITED STATES

## Abstract

The intrinsic mechanisms that promote the degeneration of retinal ganglion cells (RGCs) following the activation of N-Methyl-D-aspartic acid-type glutamate receptors (NMDARs) are unclear. In this study, we have investigated the role of downstream regulatory element antagonist modulator (DREAM) in NMDA-mediated degeneration of the retina. NMDA, phosphate-buffered saline (PBS), and MK801 were injected into the vitreous humor of C57BL/6 mice. At 12, 24, and 48 hours after injection, expression of DREAM in the retina was determined by immunohistochemistry, western blot analysis, and electrophoretic mobility-shift assay (EMSA). Apoptotic death of cells in the retina was determined by terminal deoxynucleotidyl transferace dUTP nick end labeling (TUNEL) assays. Degeneration of RGCs in cross sections and in whole mount retinas was determined by using antibodies against Tuj1 and Brn3a respectively. Degeneration of amacrine cells and bipolar cells was determined by using antibodies against calretinin and protein kinase C (PKC)-alpha respectively. DREAM was expressed constitutively in RGCs, amacrine cells, bipolar cells, as well as in the inner plexiform layer (IPL). NMDA promoted a progressive decrease in DREAM levels in all three cell types over time, and at 48 h after NMDA-treatment very low DREAM levels were evident in the IPL only. DREAM expression in retinal nuclear proteins was decreased progressively after NMDA-treatment, and correlated with its decreased binding to the c-*fos*-DRE oligonucleotides. A decrease in DREAM expression correlated significantly with apoptotic death of RGCs, amacrine cells and bipolar cells. Treatment of eyes with NMDA antagonist MK801, restored DREAM expression to almost normal levels in the retina, and significantly decreased NMDA-mediated apoptotic death of RGCs, amacrine cells, and bipolar cells. Results presented in this study show for the first time that down-regulation of DREAM promotes the degeneration of RGCs, amacrine cells, and bipolar cells.

## Introduction

Activation of NMDA-type glutamate receptors (NMDARs) plays a pivotal role in synaptic transmission by allowing calcium entry into the neuronal cells [1]. However, over-activation of NMDARs leads to a rise in intracellular calcium levels and promotes the degeneration of neuronal cells in the central nervous system (CNS), as well as in the retina [2]. In support of this, a number of previous studies have documented that the activation NMDARs increases calcium influx and promote apoptotic death of RGCs, as well as of other neuronal cells in the retina [3,4,5,6,7,8,9,10,11,12,13,14]. However, the intrinsic mechanisms that promote the degeneration of RGCs following the activation of NMDARs are still unclear.

Previous studies have reported that a rise in intracellular calcium leads to the modulation of a variety of target genes, and neuronal calcium sensing (NCS) proteins play an important role in this process [1]. Four NCS proteins that belong to a group of K-channel interacting proteins 1 to 4 (KChIP-1 to -4) have been identified to date in the CNS. A member of this family, DREAM also known as calsenilin or KChIP-3, found to be expressed widely in sensory neurons in the CNS, where its high affinity binding to DRE (downstream regulatory element) sequences represses c-*fos*- mediated expression of downstream target genes [15]. Although the function(s) of DREAM and its target genes are not completely understood, previous studies have shown that knockdown of DREAM increased NMDA-induced neuronal toxicity, while overexpression of DREAM offered neuroprotection [16]. However, the role of DREAM in the retina under normal physiological conditions or following the over-activation of NMDARs has not been reported. Therefore, this study was designed to investigate whether DREAM is expressed in the retina, and whether the expression of DREAM plays a role in NMDA-mediated degeneration of retinal neurons.

## Materials

NMDA was obtained from Sigma Chemical Company (St. Louis, MO). MK801 was procured from Tocris (Ellisville, MO). Antibody against Tuj1 (neuronal class III beta-tubulin; cat# PRB-435P) was obtained from Covance (Dedham, MA), and antibodies against PKC-alpha (cat# sc-208), Brn3a (cat# sc-31984), and DREAM (cat# sc-9142) were obtained from Santa Cruz Biotechnology (Santa Cruz, CA). Antibody against PKC-alpha was obtained from Millipore (Temecula, CA). ECL western blot substrate was obtained from Thermo Scientific (Rockford, IL). MK801 was obtained from Tocris (Minneapolis, MN).

## Methods

### Intravitreal injections

Experiments on mice were performed under general anesthesia, according to Oakland University’s institutional animal care and use committee (IACUC), which approved this study. Normal adult C57BL/6 mice (6–8 weeks old) were anesthetized by intraperitoneal injection of Ketamine (50 mg/kg body weight) and Xylazine (8 mg/kg body weight). After anesthesia, NMDA (200 nM/ 2 μL) [2,17,18] was injected into the vitreous humor of right eyes (three cohorts of six, n = 18). Left eyes received 2 μL of Phosphate-buffered saline (PBS). In separate sets of experiments, eyes (three cohorts of six, n = 18) were injected with PBS or NMDA along with MK801 (400 nM) [19,20,21].

### Extraction of nuclear proteins

At 12, 24, and 48 h after intravitreal injection, mice were euthanized with an overdose of carbon dioxide, and their eyes enucleated. Retinas from PBS- (controls) or NMDA-injected eyes were carefully removed and washed three times with PBS. Three to four retinas each were placed in Eppendorf tubes and nuclear proteins were extracted by using NE-PER Nuclear and Cytoplasmic extraction kit according to the manufacturer’s instructions (Thermo Scientific, Rockford, IL). Protein concentration in the nuclear fractions was determined by using Bio-Rad protein assay kit (Bio-Rad Laboratories, Hercules, CA).

### Western blot analysis

Aliquots containing an equal amount of retinal proteins (50 micrograms) extracted from PBS or NMDA-treated eyes were subjected to SDS-polyacrylamide electrophoresis. After electrophoresis, proteins were transferred to PVDF membranes and incubated with primary antibodies against DREAM (1:1000 dilution) or TATA binding proteins (1:1000 dilution). Membranes were washed and incubated with appropriate secondary antibody (1:2500 dilution) conjugated to horseradish peroxidase (HRP). After exposing the membranes to ECL substrate, protein bands were captured on a X-ray film.

### Electrophoretic mobility shift assay (EMSA)

Binding of DREAM to c-*fos*-DRE nucleotides was determined by EMSA, according to the general methods published previously [22,23] Briefly, aliquots containing an equal amount of nuclear proteins (10 μg) were incubated with 16 fmol of ^32^P-labeled c-*fos*-DRE (5-CTGCAGCGAGCAACTGAGAATCCAAGAC-3’) for 15 min at 37 C. Two to three mg of poly (dI-dC) was included in the binding buffer (25 mM HEPES, pH 7.9, 0.5 mM EDTA, 0.5 mM dithiothreitol, 1% Nonidet P-40, 5% glycerol, 50 mM NaCl) to inhibit non-specific binding. The DRE and DREAM complexes were separated on 7.5% native polyacrylamide gels. After electrophoresis, the gels were dried, and DRE and DREAM complexes were visualized by capturing the bands on an x-ray film.

### Immunohistochemistry

#### (A) retinal cross sections

At 12, 24, and 48 h after injecting NMDA or PBS, eyes were enucleated (three cohorts of six, n = 18), fixed in 4% paraformaldehyde, embedded in OCT compound, and ten micron-thick retinal cross sections were prepared according to the methods published previously [24]. Retinal cross sections were immunostained with antibodies (1:100 dilution) against DREAM, Tuj1, Calretinin, and PKC-alpha, and a secondary antibody (1:200 dilution) conjugated to either biotin (Vector Laboratories, Burlingame, CA) or AlexaFlour-568 and 488 (Invitrogen, Carlsbad, CA). The number of Calretinin and PKC-alpha-positive cells in retinal cross sections was quantified from photomicrographs of four microscopic fields of identical size, equal distance from the optic disc.

#### (B) Quantitation of RGCs in whole retinas

At 12, 24, and 48 h after treating the eyes with NMDA or PBS, whole retinas were isolated (three cohorts of six, n = 18) and immunostained with antibody against Brn3a (1:100 dilution) according to the methods published from this laboratory [25]. Digitized images of the whole retina was obtained by using a Zeiss Imager.Z2 microscope and compiled by using Adobe Photoshop Software 7.0 (Adobe Systems Incorporated, CA). The number of Brn3a-positive cells in each retina was quantitated in four areas of equal size from the optic disc (Boxed areas, 900 x 800 um size, 20x magnification) by using Nikon Elements AR software (Nikon Instruments Inc., Melville, NY). Statistical significance was determined by ANOVA, followed by a post hoc-Tukey’s test (GB-Stat Software, Dynamic Microsystems, Silver Spring, MD) and the results were expressed as the mean ± SEM.

### TUNEL assays

Apoptotic death of retinal neurons was determined by a TdT-mediated dUTP nick-end labeling (TUNEL) assay as previously described [24].

## Results

### Expression of DREAM in the retina

**Fig 1:**
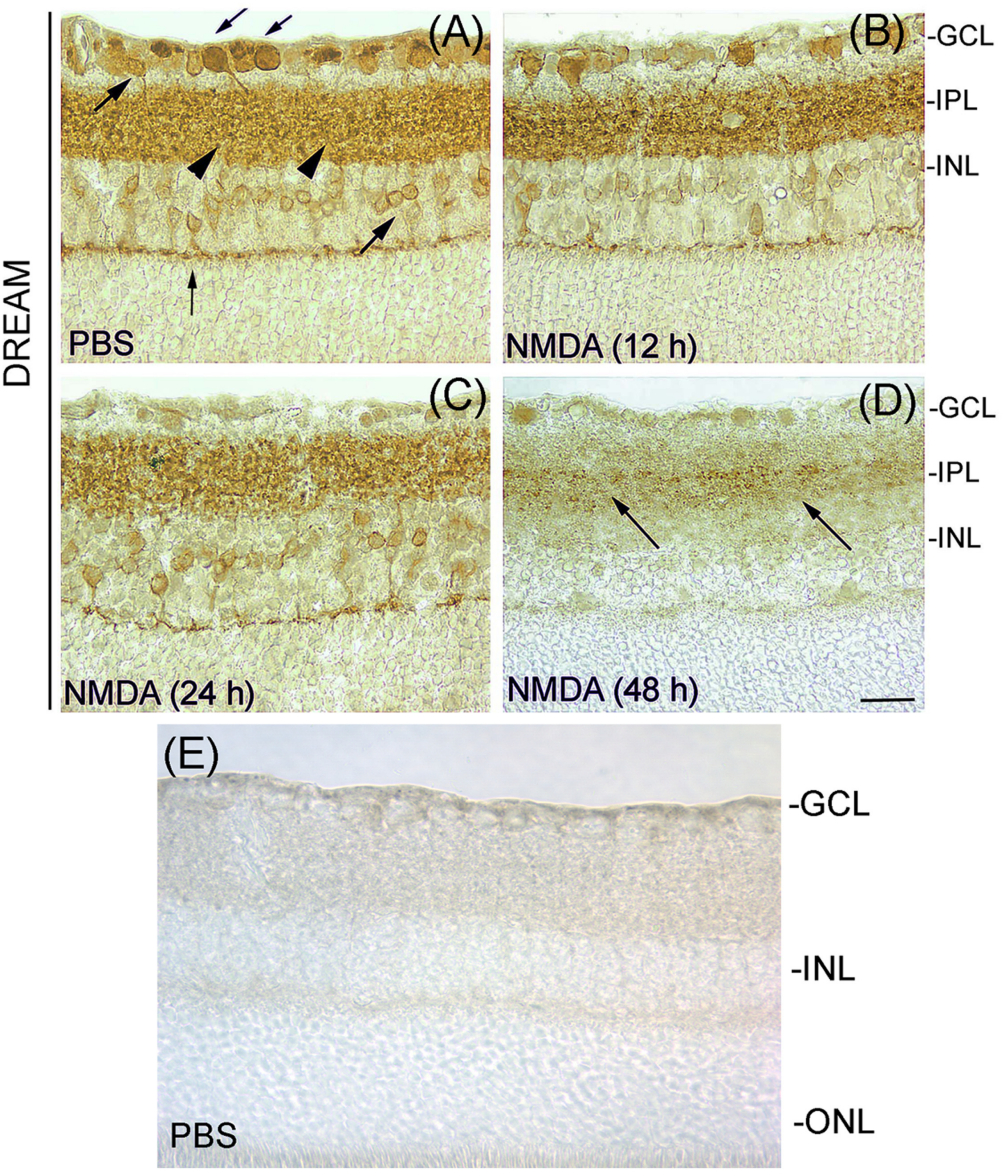
Expression of DREAM in the retina. Retinal cross sections prepared after treating the eyes with PBS or NMDA were immunostained with antibody against DREAM. Results presented in panel A show that DREAM was expressed in RGCs (down word arrows), amacrine cells (up word arrows), bipolar cells (vertical arrow), as well as in the IPL (arrow heads) in retinal cross sections prepared from PBS-treated eyes. DREAM expression was decreased following NMDA-treatment (panels B and C), and at 48 h after the treatment, very low DREAM levels were detectable in the IPL (panel D). Panel E, negative isotype control. Bar indicates 50 microns size.

To determine the cellular localization of DREAM in the retina, cross sections prepared from PBS- or NMDA-treated eyes (three cohorts of six, n = 18) were immunostained with primary antibody against DREAM and a DAB antibody detection kit. Results presented in Fig 1, panel (A) indicate that under normal physiological conditions, DREAM is expressed constitutively in the GCL, IPL, and INL. At 12 and 24 h after NMDA-treatment, DREAM expression was decreased initially in the GCL [Fig 1, panel (B)] followed by its decrease in the IPL and INL (Fig 1, panels C and D). At 48 h after NMDA-treatment, DREAM expression was decreased further in the GCL and INL, its expression was barely localized in the IPL (Fig 1, panel D). Isotype negative control shows no DREAM-positive immunostaining in the retina (Fig 1, panel E).

### NMDA down-regulates DREAM expression in the retina

**Fig 2:**
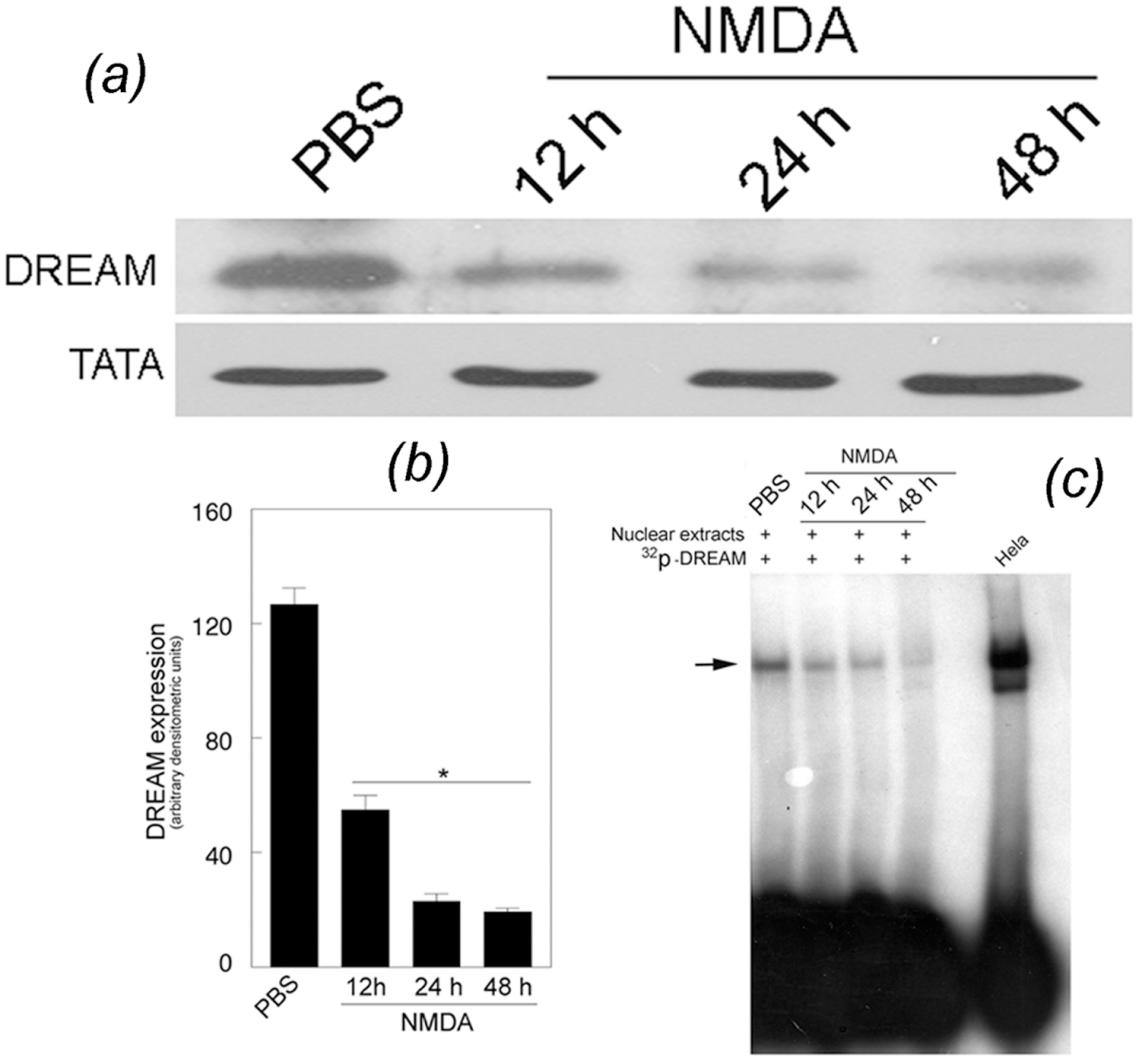
Relative levels of DREAM in the retina. (*a*) Western blot analysis performed by using antibodies against DREAM and TATA binding protein indicate that DREAM expressed constitutively in retinal nuclear proteins extracted from PBS-treated eyes was reduced in proteins extracted from NMDA-treated eyes. (*b*) Semi-quantitative analysis of western blots indicate a significant decrease in DREAM levels in retinal protein extracted from NMDA-treated eyes when compared to PBS-treated eyes. *p<0.05. (*c*) EMSA assays show that the binding of DREAM to DRE oligonucleotides was decreased in retinal nuclear proteins extracted from NMDA-treated eyes (horizontal arrow), when compared to PBS-treated eyes.

Since the above immunohistochemical analyses indicated that NMDA decreased DREAM levels in the GCL, INL, and IPL, western blot analysis was performed to determine the relative levels of DREAM in nuclear proteins extracted from the retinas of eyes treated with PBS or NMDA (three cohorts of six, n = 18). Results presented in Fig 2(*a*) indicate that the levels of DREAM, observed constitutively in the nuclear proteins extracted from PBS-treated eyes, were decreased progressively in MMDA-treated eyes at 12, 24, and 48 h after the treatment [Fig 2(*a*)]. Semi-quantitative analysis also indicate that the DREAM levels were decreased significantly in retinal nuclear proteins extracted from NMDA-treated eyes [Fig 2(*b*)], when compared to the retinal nuclear proteins extracted from PBS-treated eyes (*, ** p<0.05). EMSA assays indicate that the binding of DREAM to c-*fos*-DRE oligonucleotides was decreased in retinal nuclear proteins extracted from NMDA-treated eyes, when compared to the nuclear proteins extracted from PBS-treated eyes [Fig 2(*c*)]. *,*p<0.05.

### Down-regulation of DREAM correlates with apoptotic death of retinal neurons

**Fig 3:**
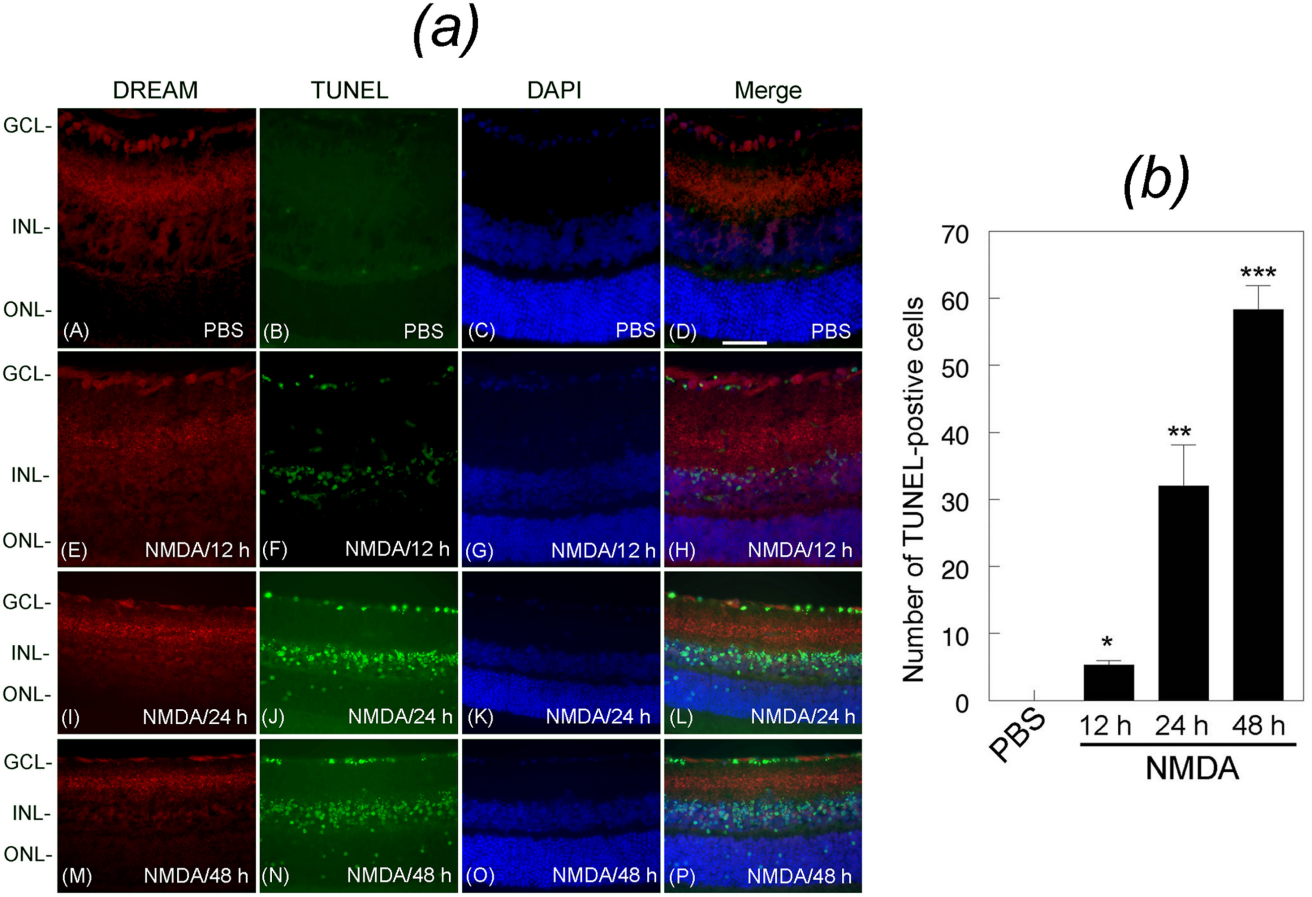
Expression of DREAM and apoptotic cell death in the retina. (*a*) Retinal cross sections prepared from PBS or NMDA-treated eyes were immunostained with antibody against DREAM (panels A, E, I, and M), subjected to TUNEL assays (panels B, F, J, and N), counter stained with DAPI (panels C, G, K, and O), and the images were merged (D, H, L, and P). Results presented in the figure show that DREAM was expressed in the GCL, IPL, and INL in retinal cross sections prepared from PBS-treated eyes (panels A and D). DREAM expression was decreased at 12 h (panels E and H), 24 h (panels I and L), and 48 h (panels M and P) after NMDA treatment. No TUNEL-positive cells were observed in retinal cross sections prepared from PBS-treated eyes (panel B), but increased number of TUNEL-positive cells were observed in retinal cross sections prepared from NMDA-treated eyes (panels F, J, N). Merged images indicate that TUNEL-positive cells were localized initially in the GCL (panel H), and later in the INL (panel L) and the ONL (panel P). Bar indicates 50 microns size. (*b*) Quantitative analysis indicates that when compared to PBS treatment, NMDA treatment promoted apoptotic cell death significantly over a 48 h time period,.*, **, *** p<0.05.

To determine whether NMDA-mediated down-regulation of DREAM promotes apoptotic death of neuronal cells in the retina, cross sections prepared from PBS or NMDA-injected eyes (three cohorts of six, n = 18) were immunostained with antibody against DREAM protein followed by the detection of apoptotic cells by performing TUNEL assays. Results presented in Fig 3(*a*) indicate that DREAM was expressed in the GCL, IPL, and INL consistent with the results presented in Fig 1. TUNEL assays indicate that at 12 h after NMDA treatment, DREAM expression was down-regulated in a few cells in the GCL [Fig 3(*a*), panel E], and those cells that expressed reduced levels of DREAM started to undergo apoptotic cell death [Fig 3(*a*), panel F]. At 24 h and 48 h after NMDA-treatment, DREAM was reduced further in the GCL and in the INL [Fig 3(*a*), panels I and M], and this decrease also correlated with increased number of TUNEL-positive cells [Fig 3(*a*), J and N]. Quantitative analysis of TUNEL-positive cells indicate that NMDA promoted significant death of retinal neurons over a 48 h time period [Fig 3(*b*)]. *,**,***p<0.05.

### Down-regulation of DREAM promotes the degeneration of RGCs, amacrine cells, and bipolar cells

To determine the cell types that degenerated following NMDA-treatment, experiments were performed further by using antibodies specific to RGCs, amacrine cells, and bipolar cells.

**Fig 4:**
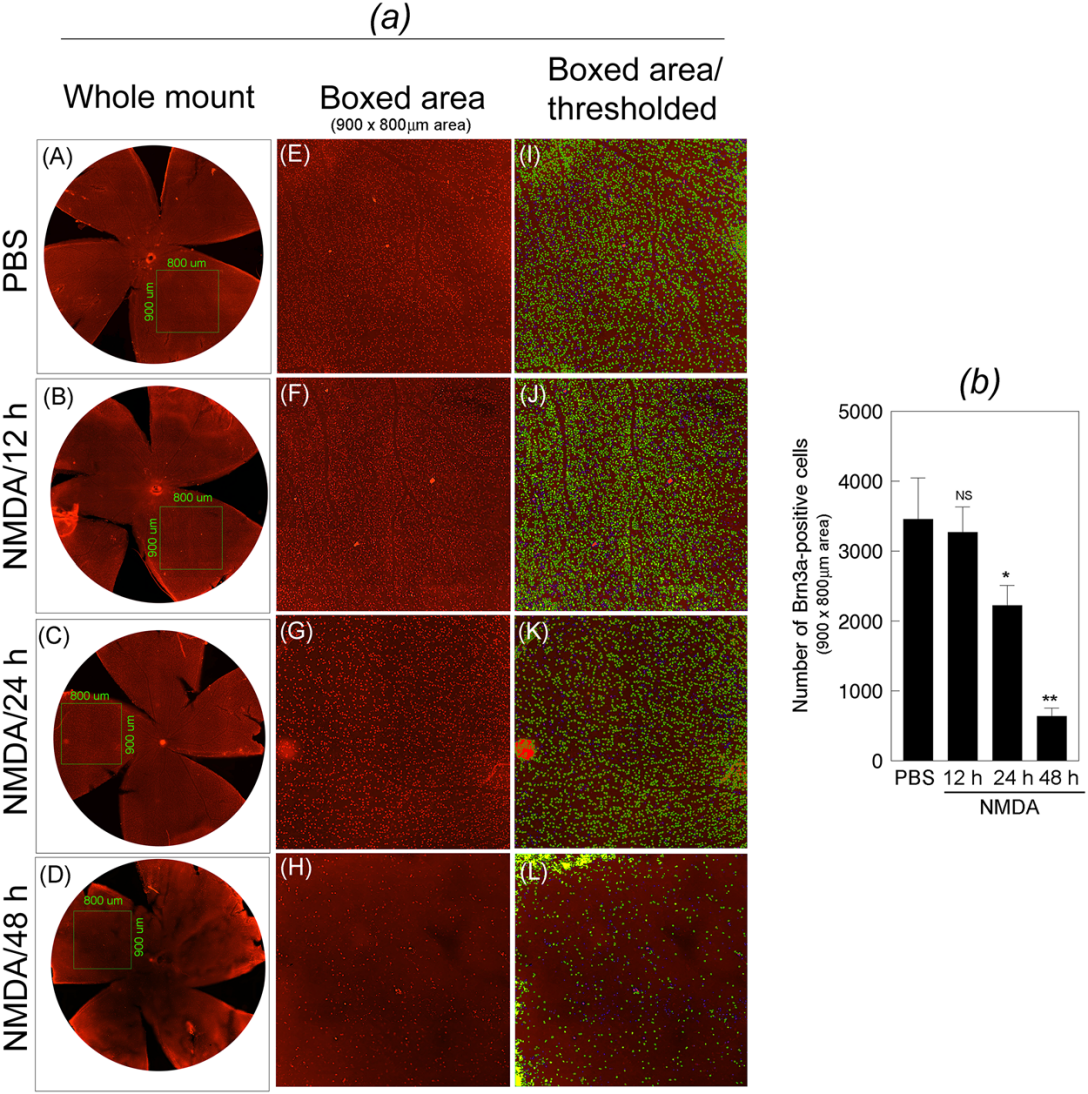
Characterization of RGCs’ loss in the retina. (*a*) After treating the eyes with PBS (panel A) or NMDA, whole retinas were isolated at 12 h (panel B), 24 h (panel C), and 48 h (panel D), and immunostained with antibody against Brn3a. From each retina, the number of Brn3a-positive RGCs in the boxed areas (four areas per retina) of equal size (900 x 800 um size; panels E, F, G, and H) were quantified by adjusting the threshold of the images (panels I, J, K, and L) and by using Nikon elements AR software. Results presented in the figure indicate that when compared to the Brn3a-positive RGCs in the retinas isolated from PBS-treated eyes (panels A, E, I), the number of Brn3a-positive RGCs in the retinas isolated at 12 h (panels B, F, J), 24 h (panels C, G, K), and 48 h (panels D, H, L) after NMDA-treated eyes was decreased progressively over time. (*b*) Quantification of the Brn3a-positive cells indicate that when compared to PBS treatment, NMDA treatment significantly decreased the number of RGCs,. *, **p<0.05. NS, not significant.

To determine the loss of RGCs, mouse eyes (three cohorts of six, n = 18) were injected with NMDA or PBS, and at 12, 24, and 48 h after the treatment, whole retinas were isolated and immunostained with antibody against Brn3a. Results presented in Fig 4(*a*), left most panels (whole mount retinas) and areas of equal size from the optic disc (Boxed areas, 900 x 800 um size, 20x magnification) indicate that a reduction in DREAM levels (Figs 1 and 2) correlated with a progressive loss of RGCs over a 48 h time period. The number of Brn3a-positive cells in boxed area of equal size were estimated by adjusting the threshold for the size and intensity. Quantitative data presented in Fig 4(*b*) indicate that NMDA promoted significant degeneration of RGCs. *,**p<0.05.

**Fig 5:**
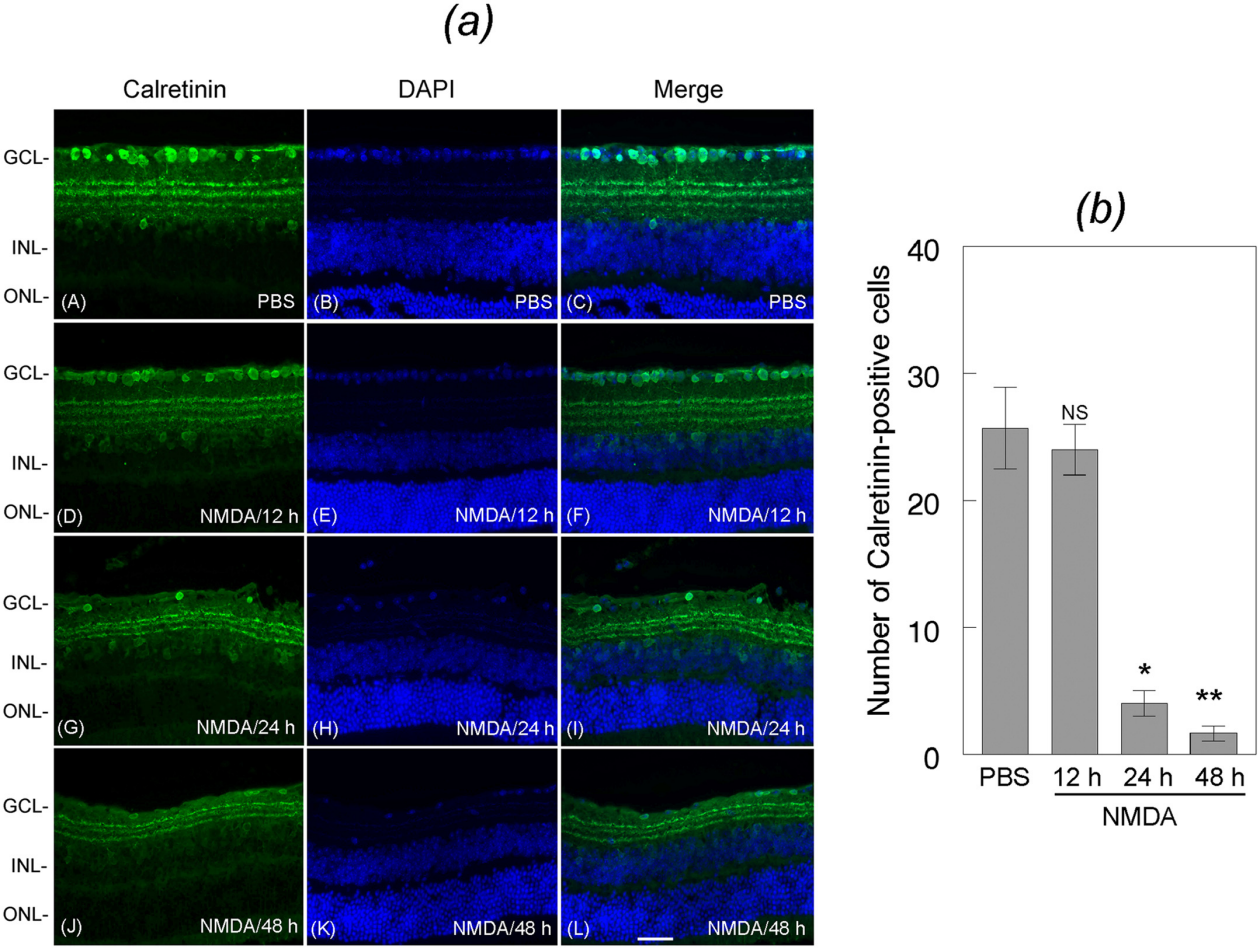
Characterization of the loss of amacrine cells. (*a*) Retinal cross sections prepared at 12 h, 24 h, and 48 h after treating the eyes with NMDA or PBS were immunostained with antibody against calretinin, and their number was quantified (*b*). Results presented in the figure indicate that when compared to the Calretinin-positive amacrine cells in retinal cross sections prepared from PBS-treated eyes (panels A, B, C), the number of amacrine cells was reduced in the retinal cross sections prepared from NMDA-treated eyes at 12 h (panels D, E, F), 24 h (panels G, H, I), and 48 h (panels J, K, L) after the treatment. (*b*) Quantification of the calretinin-positive cells indicate that the number of amacrine cells was significantly reduced after NMDA-treatment. *, **p<0.05. NS, not significant. Bar indicates 50 microns size.

**Fig 6:**
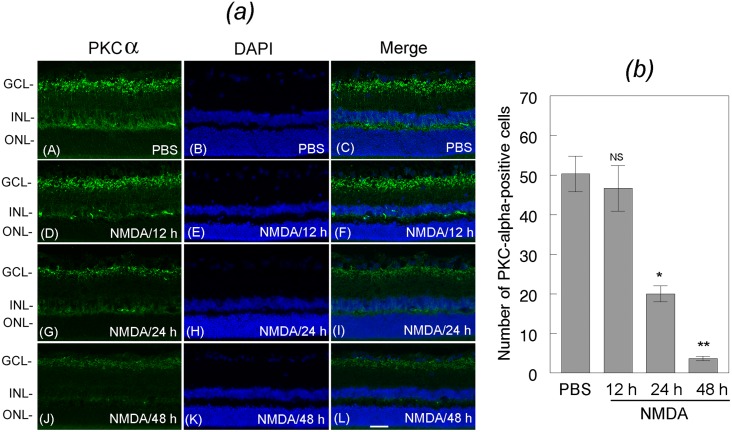
Characterization of the loss of bipolar cells. (*a*) Retinal cross sections prepared at 12 h, 24 h, and 48 h after treating the eyes with NMDA or PBS were immunostained with antibody against PKC-alpha, and their number was quantified (*b*). Results presented in the figure indicate that when compared to the PKC-alpha-positive bipolar cells in retinal cross sections prepared from PBS-treated eyes (panels A, B, C), the number of bipolar cells was decreased in retinal cross sections prepared from NMDA-treated eyes at 12 h (panels D, E, F), 24 h (panels G, H, I), and 48 h (panels J, K, L) after the treatment. (*b*) Quantification of the PKC-alpha-positive cells indicate that the number of bipolar cells was significantly reduced after NMDA-treatment. *, **p<0.05. NS, not significant. Bar indicates 50 microns size.

To determine whether loss of DREAM promotes the loss of amacrine cells and bipolar cells, mouse eyes (three cohorts of six, n = 18) were treated with NMDA or PBS. At 12, 24, and 48 h after the treatment, retinal cross sections were immunostained with antibodies against Calretinin, a marker for amacrine cells (Fig 5) and PKC-alpha, a marker for bipolar cells (Fig 6). Results presented in Fig 5(*a*) indicate that the number of Calretinin-positive amacrine cells was decreased progressively in retinal cross sections prepared from NMDA-treated eyes, but not in PBS-treated eyes. Semi-quantitative analysis indicate a significant loss of amacrine cells over a 48 h period time [Fig 5(*b*)]. *,**p<0.05. Results presented in Fig 6(*a*) indicate that the number of PKC-alpha-positive bipolar cells also decreased in retinal cross sections prepared from NMDA-treated eyes at 24 [Fig 6(*a*), panel G] and 48 h [Fig 6(*b*), panel J], when compared to PBS-treated eyes [Fig 6(*b*), panel A]. Semi-quantitative data presented in 6 (*b*) indicate that the NMDA-mediated decrease in DREAM expression (Figs 1 and 2) correlate significantly with the loss of bipolar cells at 24 h and 48 h after the treatment. *,**p<0.05.

### NMDA-receptor antagonist, MK801, attenuates the loss of DREAM

**Fig 7:**
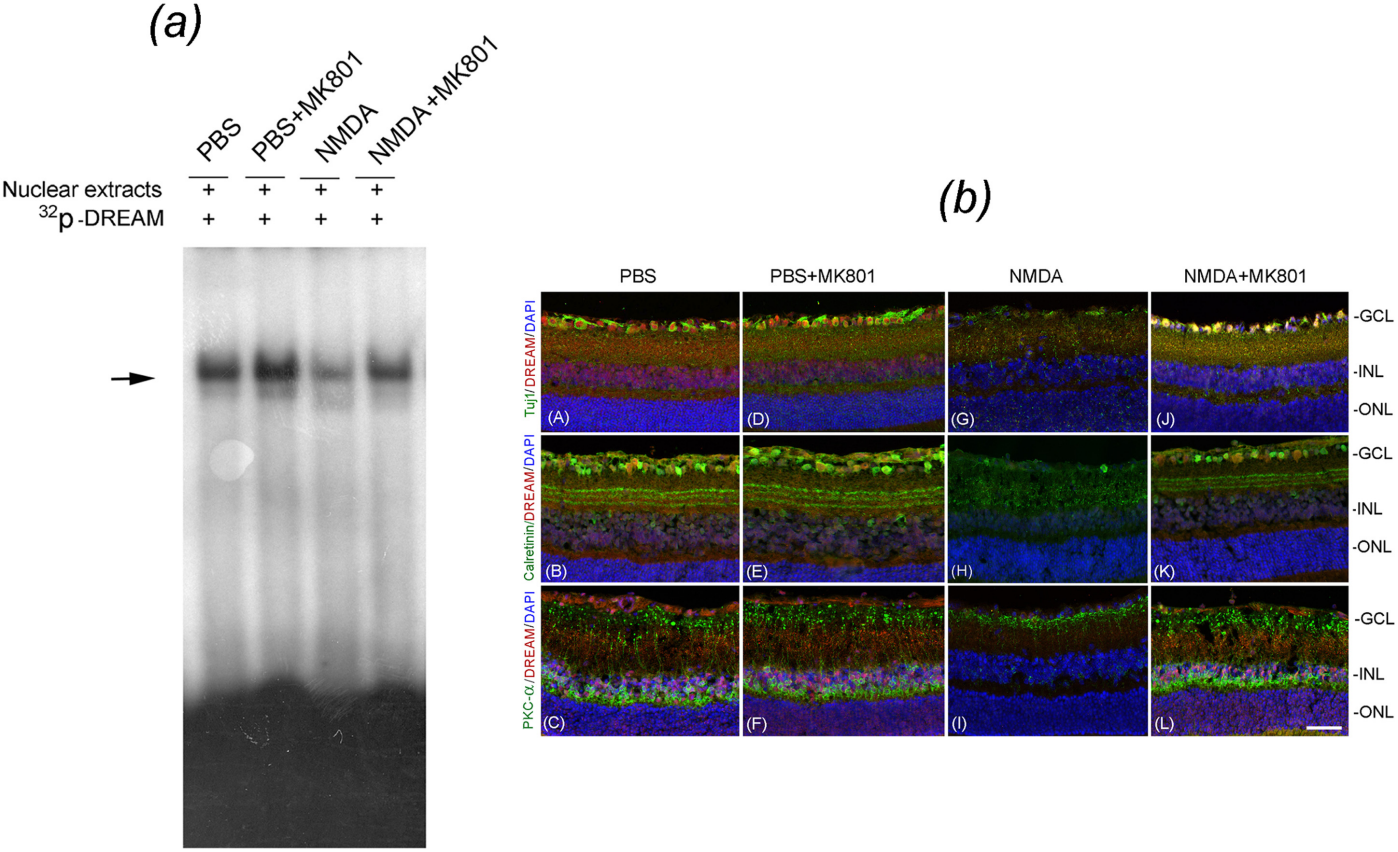
Effect of MK801 on DREAM expression and NMDA-induced cell loss in the retina. (*a*) EMSA assays preformed on nuclear proteins extracted from the retinas at 24 h after treating the eyes with PBS, PBS plus MK801, NMDA, and NMDA plus MK801 indicate that the binding of DREAM to DRE oligonucleotides was decreased in retinal nuclear proteins extracted from NMDA-treated eyes [panel (*a*), horizontal arrow, third lane], when compared to PBS-treated eyes [panel (*a*), horizontal arrow, first lane]. In contrast, MK801 restored NMDA-mediated decrease of DREAM binding to DRE nucleotides [panel (*a*), fourth lane]. (*b*) Retinal cross sections prepared 24 h after treating the eyes with PBS, PBS plus MK801, NMDA, and NMDA plus MK801 were subjected to immunohistochemistry by using antibodies against DREAM along with Tuj1, Calretinin, and PKC-alpha, and counter-stained with DAPI. Results presented the figure indicate that the DREAM (red fluorescence), co-localized with Tuj1-positive RGCs (green fluorescence) in retinal cross sections prepared from PBS-treated eyes [Fig. (*b*), panel (A)] was decreased in NMDA-treated eyes and associated with the loss of RGCs [Fig. (*b*), panel (G)]. In contrast, treatment of the eyes with NMDA and MK801 prevented the loss of DREAM and the degeneration of RGCs [Fig. (*b*), panel (J)]. In addition, DREAM protein (red fluorescence), co-localized with Calretinin-positive amacrine cells (green fluorescence) in retinal cross sections prepared from PBS-treated eyes [Fig. (*b*), panel (B)] was reduced in NMDA-treated eyes and associated with the degeneration of amacrine cells [Fig. (*b*), panel (H)]. In contrast, treatment of the eyes with NMDA and MK801 attenuated the loss of DREAM and the degeneration of amacrine cells [Fig. (*b*), panel (K)]. Furthermore, DREAM (red fluorescence) expressed in PKC-positive bipolar cells (green fluorescence) was also reduced in retinal cross sections prepared from NMDA-treated eyes and associated with the degeneration of bipolar cells [Fig. (*b*), panel (I)]. However, treatment of the eyes with NMDA and MK801 attenuated the loss of DREAM and NMDA-mediated degeneration of bipolar cells [Fig. (*b*), panel (L)]. Bar indicates 50 microns size.

To determine whether inhibition of NMDAR activation attenuates apoptotic death of retinal neurons, mouse eyes (three cohorts of six, n = 18) were treated with PBS or NMDA along with MK801. EMSA assays indicate [Fig 7(*a*)] that the binding of DREAM to DRE nucleotides was reduced in nuclear proteins extracted from NMDA-treated eyes, when compared to PBS-treated eyes. In contrast, MK801 restored the binding of DREAM to DRE oligonucleotides even in the presence of NMDA. MK801 had no effect on DREAM binding to DRE nucleotides in nuclear proteins extracted from PBS-treated eyes.

Since MK801 attenuated the loss of DREAM in retinal protein extracts [Fig 7(*b*)], experiments (three cohorts of six, n = 18) were performed to further determine whether MK801 prevents the loss of RGCs, amacrine cells, and bipolar cells by using antibodies against Tuj1, Calretinin, and PKC-alpha respectively. Results presented in Fig 7(*b*) indicate that DREAM was expressed in Tuj1-positive RGCs (panel A), Calretinin-positive amacrine cells (panel B), and PKC-alpha-positive bipolar cells (panel C) in retinal cross sections prepared from PBS-treated eyes. In contrast, at 48 h after NMDA treatment, expression of DREAM is decreased considerably and correlated with the degeneration of RGCs (panel G), amacrine cells (panel H), and bipolar cells (panel I). Interestingly, retinal cross sections prepared after treating the mice eyes with NMDA plus MK801, DREAM expression was restored in RGCs (panel J), amacrine cells (panel K), and bipolar cells (panel L), and attenuated their degeneration.

### MK801 attenuates apoptotic death of retinal neurons

**Fig 8:**
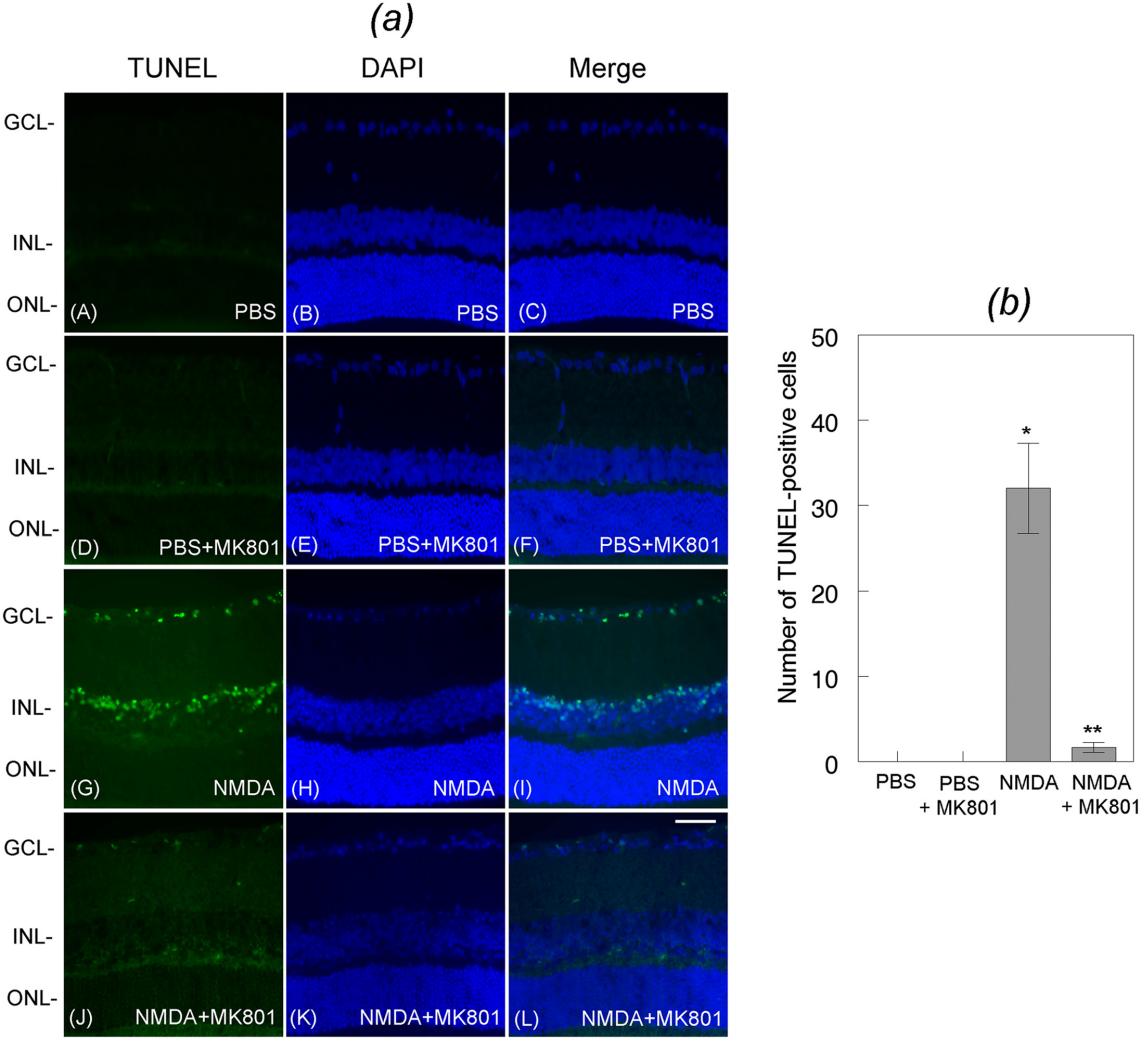
Effect of MK801 on NMDA-mediated apoptotic death of retinal neurons. Retinal cross sections prepared from PBS or NMDA-treated eyes were subjected to TUNEL assays. Results presented in the figure indicate that when compared to PBS [Fig. (*a*), panels A, B, C], NMDA promoted significant apoptotic cell death in the GCL and the INL at 24 h after the treatment [Fig. (a), panels G, H, I]. In contrast, treatment of the eyes with NMDA and MK801 significantly attenuated NMDA-mediated apoptotic death of cells both in the GCL and INL [Fig. (*b*)]. Bar indicates 50 microns size. Characterization of neuronal cell’ loss in whole retinas and in retinal cross sections indicates that NMDA promoted a significant loss (*p<0.05) of Brn3a-positive RGCs (C), calretinin-positive amacrine cells (D), and PKC-alpha-positive bipolar cells (E), and MK801 attenuated the loss of all three cell types (**p<0.05).

**Fig 9:**
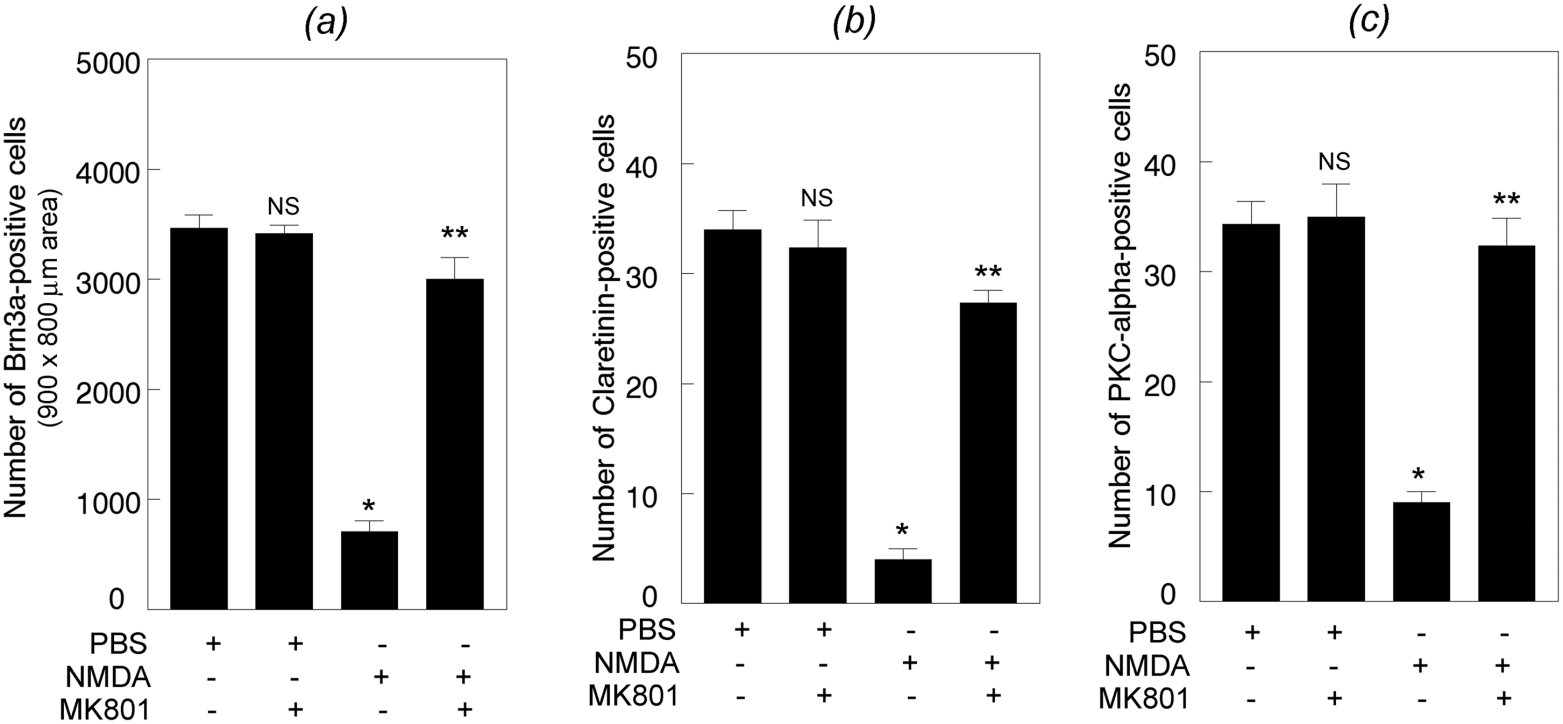
Effect of MK801 on NMDA-induced loss of retinal neurons. Whole retinas (panel A) or retinal cross sections (panels B and C) prepared 24 h after treating the eyes with PBS, PBS plus MK801, NMDA, and NMDA plus MK801 were immunostained with antibodies against Brn3a (panel A), Calretinin (panel B), and PKC-alpha (panel C), quantified by using Nikon elements AR software. Results presented in the figure indicate that NMDA promoted significant loss of Brn3a-positve RGCs, Calretinin-positive amacrine cells, and PKC-alpha-positive bipolar cells. *p<0.05. In contrast, treatment of eyes with NMDA along with MK801 significantly attenuated the degeneration of all three cell types. **p<0.05. NS, not significant.

Since MK801 restored DREAM expression, additional experiments were performed to investigate whether restored expression of DREAM attenuates apoptotic death of retinal neurons. Mouse eyes (three cohorts of six, n = 18) were treated with PBS or NMDA along with MK801 and retinal sections prepared at 24 h after the treatment were subjected to TUNEL assays. Results presented in Fig 8(*a*) show no apoptotic cells in retinal cross sections prepared from PBS (panels A-C) or PBS plus MK801-treated eyes (panels D-F). In contrast, increased number of TUNEL-positive cells were observed in retinal cross sections prepared at 24 h after NMDA-treatment (panels G-I). Interestingly, apoptotic cell death was decreased in retinal cross sections prepared from the eyes treated with NMDA along with MK801 (panels J-L). Quantitative analysis indicate that NMDA-mediated apoptotic death was significantly reduced in retinal cross sections prepared from NMDA plus MK801-treated eyes [Fig 8(*b*)]. *,**p<0.05. Quantification of the loss of neuronal cells by using antibodies against Brn3a, Calretinin, and PKC-alpha indicate that when compared to PBS, NMDA induced a significant degeneration of Brn3a-positive RGCs [Fig 9, panel (*a*)], Calretinin-positive amacrine cells [Fig 9, panel (*b*)], and PKC-alpha-positive bipolar cells [Fig 9, panel (*c*)]. *p<0.05, when compared to PBS-treatment; **p<0.05, when compared to NMDA-treatment.

## Discussion

Although a few studies on the CNS have reported that DREAM functions as a negative modulator of NMDA-mediated neuronal damage [16,26] until now the role of DREAM in the retina under normal conditions or following NMDA-induced retinal damage has not been investigated. In this study, we have shown that DREAM is expressed in RGCs, amacrine cells, and bipolar cells under normal physiological conditions and that DREAM plays a major role in the survival of these cells. In support of this statement, we have shown that NMDA led to a significant decrease in the expression of the DREAM in all three cell types, and a decrease in DREAM in turn, promoted apoptotic death of RGCs, amacrine cells, and bipolar cells. Remarkably, inhibition of the activation of NMDARs by MK801 restored the expression of the DREAM significantly in RGCs, amacrine cells, and bipolar cells, and attenuated apoptotic death of all three-cell types. Thus, the results presented in this study are important steps forward towards understanding the mechanisms underlying the degeneration of retina following the activation of NMDARs.

First, a few studies have reported contradicting results regarding the role of DREAM in the retina. For example, previous studies have reported that the activation of NMDARs decreased the expression of the DREAM [27], while hyperglycemia up-regulated the expression of the same protein in cultured Müller cells [28]. Nonetheless, it is unclear how DREAM expressed in Müller cells affects the survival of RGCs and other cell types. Therefore, the results presented in this study are important and provide concrete evidence that the DREAM is indeed expressed not only in RGCs, but also in amacrine cells and bipolar cells, and NMDA-mediated down-regulation of DREAM promoted apoptotic death of all three-cell types.

Second, the intrinsic proteins/factors that promote NMDA-mediated degeneration of RGCs have not been identified. Previous studies [5,29] including studies from our laboratory [30,31] have shown that over stimulation of glutamate receptors promoted the degeneration of RGCs by modulating extrinsic signals. For example, we have reported that over-stimulation of glutamate receptors promoted the degeneration of RGC by up-regulating the expression of extracellular matrix (ECM)-degrading proteases. Although several studies from other laboratories have reported that the activation of NMDARs up-regulated the expression of phospho-p38 and phospho-Akt in the GCL and in the INL, it was unclear how these intrinsic signals promoted the degeneration of RGCs [2]. Finally, a recent study reported that the activation of NMDARs promoted the degeneration of RGCs by down-regulating the expression another intrinsic protein, Apolipoprotein E (ApoE), in astrocytes and Müller cells in the mouse retina [32], but it is still unclear how ApoE that is expressed in glial cells promote the degeneration of RGCs. In this study, we have shown that activation of NMDARs down-regulated the expression of the intrinsic protein DREAM in RGCs, amacrine cells, and bipolar cells (not in supportive glial cells), and down-regulation of DREAM, in turn, leads to apoptotic death of these cells.

An interesting question that needs to be addressed is why DREAM, a calcium sensing protein supposed to be expressed constitutively in the nucleus, is expressed not only in the nucleus, but also in the synaptic layer of the INL. Currently, we do not have sufficient scientific data to explain this observation, but we believe that under normal physiological conditions neuronal cells, such as RGCs, amacrine cells, and bipolar cells do not have sufficient time to synthesize DREAM to repress c-*fos*-mediated genes whether they are essential or detrimental. Thus, we propose that under normal physiological conditions, depending on the levels of calcium these cells express DREAM constitutively, and dynamically transport it between the cytoplasm and the nucleus to regulate the expression of target genes. In summary, the results presented in this study show that intrinsically expressed DREAM is needed for the survival of RGCs, amacrine cells, and bipolar cells.
